# Therapeutic targeting of lysosome-triggered inflammatory channels in nasal and upper-airway allergic conditions

**DOI:** 10.3389/fimmu.2026.1799685

**Published:** 2026-05-26

**Authors:** Haoran Yu, Yujin Zheng, Daquan Wu, Lei Zhou, Kanglun Jiang, Kaisai Tian, Na Shen

**Affiliations:** 1 Department of Otorhinolaryngology-Head and Neck Surgery, ZhongShan Hospital Fudan University, Shanghai, China; 2 Department of Otorhinolaryngology-Head and Neck Surgery, ZhongShan Hospital (Xiamen), Fudan University, Xiamen, Fujian, China

**Keywords:** allergic rhinitis, immune signaling, ion channels, lysosome, NLRP3, therapeutics, upper-airway inflammation

## Abstract

Nasal and upper airway allergic disorders, such as allergic rhinitis and allergy-associated sinus inflammation, constitute a major global health problem because of their high prevalence and significant negative impact on quality of life. There are indications that lysosomes to a large extent participate in the process of airway inflammation, by both starting and intensifying the immunological signaling via certain ionic channels. The resulting release of pro-inflammatory cytokines occurs through calcium signaling mediated by lysosome activity, and this is particularly the case when the NLRP3 inflammasome is activated. Hence, mucosal inflammation and increased airway sensitivity develop. Recent studies have identified the lysosomal ion channels, such as the two-pore channels (TPCs) and transient receptor potential mucolipin (TRPML), as the main regulators of these processes. The animal models used for preclinical settings confirmed that manipulation of these channels pharmacologically or genetically had a promising anti-inflammatory effect, thus marking them as possible future therapeutic targets. Moreover, such approaches may help reduce systemic side effects and enhance the efficacy of existing treatments. This article reviews the molecular mechanisms, therapeutic strategies, and translational potential of lysosome-triggered inflammatory signaling in nasal and upper airway allergic disorders. Targeting lysosomal signaling pathways may provide new opportunities for precision-based therapies in allergic airway inflammation.

## Introduction

Nasal and upper airway allergic conditions, including allergic rhinitis (AR) and allergic sinus inflammation, are among the most prevalent disorders affecting the global population and are primarily caused by immunological reactions to environmental allergens. AR is a major global health concern due to its high prevalence and substantial burden on both patients and healthcare systems. Common symptoms, including nasal congestion, sneezing, rhinorrhea, and postnasal drip, significantly impair quality of life and contribute to increased socioeconomic costs. Although standard therapies such as antihistamines, intranasal corticosteroids, and allergen-specific immunotherapy remain the mainstay of treatment, a considerable number of patients continue to experience inadequate symptom relief or treatment-related side effects. This highlights the need for the development of novel and more targeted therapeutic approaches (1).

Traditionally, allergic inflammation of the upper respiratory tract was ascribed to the activation of mast cells through IgE antibodies, the activation of Th2-biased adaptive immune responses, and the influx of eosinophils and other inflammatory cells (2, 3). On the other hand, the shifting evidence presents intracellular organelles, especially lysosomes, as the immune signaling activators instead of the previously thought passive degradative compartments, thus changing the entire scenario (4). Lysosomes help in ion homeostasis and, if they become destabilized, they can empty their contents into the cytosol which is a series of events that might activate the inflammasome and further inflammatory cascades (5).

Among the various inflammasomes, the NLRP3 inflammasome has become the primary one to monitor and detect the cellular stress and hazard signals. NLRP3 when activated forms a complex with the adaptor proteins and the caspases and then the cleavage of pro-inflammatory cytokines like interleukin 1β (IL 1β) and IL 18 takes place which results in their secretion, and in many cases, the inflammatory cell death (pyroptosis) processes which are playing a major role in causing inflammation in the airways, are also initiated along with the cleaved pro-inflammatory cytokines (6, 7). More specifically, recent data from AR models have shown that NLRP3 activation in macrophages promotes increased IL-1β release and macrophage pyroptosis in the nasal mucosa, thereby contributing to local inflammation. In contrast, pharmacological inhibition of NLRP3 has been shown to reduce these effects, indicating its potential as a promising therapeutic target (8).

Re-evaluating lysosomes as active signaling hubs rather than merely “cellular waste bins” provides new mechanistic insights and therapeutic opportunities. Targeting lysosome-initiated signaling pathways, such as ion fluxes that lead to inflammasome activation, may enable more selective modulation of inflammation, thereby reducing reliance on broad immunosuppression and minimizing systemic side effects (9, 10). Therefore, this review aims to summarize the existing knowledge regarding the role of lysosome-triggered inflammatory pathways in nasal and upper airway allergic conditions, particularly allergic rhinitis and related inflammatory disorders. We will particularly focus on lysosome biology in immune and respiratory cells and discuss the molecular links between lysosomal ion channels, ion flux, membrane perturbation, and inflammasome activation. Moreover, the current review evidence from allergic airway disease models implicating these pathways and exploring current and future therapeutic strategies targeting lysosomal signaling with translational potential will be presented.

## Review methodology

A thorough search of published research on lysosomal ion channels, inflammasome signaling, and upper-airway allergy disorders particularly allergic rhinitis was used to perform this narrative review. Electronic databases such as PubMed, Scopus, and Google Scholar were used to find pertinent publications. Combinations of “lysosome,” “lysosomal ion channels,” “NLRP3 inflammasome,” “allergic rhinitis,” “upper airway inflammation,” “nasal allergy,” and “airway immune response” were among the search terms used.

Peer-reviewed original research publications, review articles, and current studies published within the last ten years were prioritized; nevertheless, when necessary for mechanistic understanding, seminal earlier works were also included. Research on lysosomal dysfunction, inflammasome activation, allergic airway inflammation, and translational treatment approaches was thoroughly reviewed. In order to compile the most recent data on the molecular function and prospective therapeutic applications of lysosome-triggered inflammatory pathways in nasal and upper-airway allergy disorders, the chosen literature was examined.

### Lysosome biology and immune signaling

Lysosomes have long been regarded as the “recycling centers” of the cell, primarily responsible for the degradation of macromolecules and cellular waste through the action of acidic hydrolases. However, over the past decade, growing evidence has revealed that lysosomes function as dynamic signaling hubs that play essential roles in maintaining cellular homeostasis, metabolic sensing, and immune regulation (11).

### Lysosome structure, ion homeostasis and dynamics

Lysosomes, as to their structure, are organelles that have a single membrane and their lumen that has an acidic pH (~4.5-5.5) that is required for the best activity of their hydrolytic enzymes (12). The membrane of lysosomes has various types of ion channels and transporters which are responsible for regulating the coming in and going out of different ions like H^+^, Ca²^+^, Na^+^, K^+^, and sometimes Fe²^+^ and Zn²^+^ (13).

These channels and transporters not only serve the degrading functions (acidification, enzyme activity, catabolite export) but also play a role in lysosomal signaling: ion fluxes control membrane potential, luminal ion composition, and downstream signaling cascades. For instance, Ca²^+^ which is stored in lysosomes can be quickly released into the cytosol through lysosomal Ca²^+^ channels resulting in a strong intracellular signal being generated even though the amount of ions that are released is small due to the steep concentration gradient across the lysosomal lumen (~0.5 mM Ca²^+^) and cytosol (~100 nM) (14, 15).

Lysosomes are not only dynamic in their positioning but also in their morphology and interactions with other organelles. Their trafficking such as movement along microtubules, fusion/fission events, exocytosis, and membrane contact site formation depends on proper ion channel function (16). Activation of a lysosomal calcium channel is required for calcium-dependent centripetal movement of lysosomes toward the perinuclear region under autophagy induction. Thus, by integrating ion homeostasis, trafficking, and membrane dynamics, lysosomes act as more than waste disposal compartments; they are central to cellular regulation and responsiveness (17).

### Lysosomal ion channels: types and their functions

Several ion channels are present on lysosomal and endolysosomal membranes, where they regulate ion homeostasis and support fundamental cellular processes. Among the most prominent are the transient receptor potential mucolipin (TRPML1-3) channels, which are nonselective cation channels permeable to Ca²^+^, Fe²^+^, Zn²^+^, and other ions. Their activity is regulated by phosphoinositides such as PI (3, 5)P_2_ and by luminal pH. TRPML1, the best-characterized member, is essential for lysosomal exocytosis, phagosome maturation, autophagy, membrane repair, and overall lysosomal ion homeostasis (18). Two-pore channels (TPC1 and TPC2) also contribute to endolysosomal ion regulation by mediating Na^+^ and Ca²^+^ fluxes, thereby influencing vesicular trafficking and intracellular signaling (14).

In addition, potassium channels such as TMEM175 and other K^+^-permeable conductances help maintain lysosomal membrane potential and support autophagic flux. Chloride channels and proton transport mechanisms further contribute to lysosomal acidification, charge balance, and optimal enzymatic activity. Through coordinated ion fluxes, these channels regulate lysosomal pH stability, membrane trafficking, and organelle biogenesis, thereby sustaining cellular homeostasis (19).

### Lysosomes as signaling hubs in immune cells

In immune-related cells such as macrophages, dendritic cells (DCs), and lymphocytes, lysosomes function not only as degradative organelles but also as active regulators of immune responses. They influence immune cell fate through processes such as antigen processing and presentation, cytokine production, and the regulation of cellular signaling pathways involved in inflammation and immune activation (4). Reduced lysosomal stability in DCs, for example following the phagocytosis of particulate matter, may lead to the release of cathepsins into the cytosol. This process can trigger inflammasome assembly, particularly through NLRP3 activation, resulting in the maturation and release of pro-inflammatory cytokines such as IL-1β and IL-18. Nevertheless, lysosomes also play an important role in preventing excessive inflammation. Through autophagy, they facilitate the degradation of inflammasomes and damaged mitochondria, thereby limiting the activation of inflammatory pathways and maintaining immune homeostasis (20, 21).

In the case of macrophages, the activation of the lysosomal channel TRPML1 controls inflammatory responses by regulating the discharge of lysosomal Fe²^+^ which in turn alters PHD enzymes and thus, reduces NF κB-driven transcription of inflammatory genes like IL1B (22). This finding highlights a direct mechanistic link between lysosomal ion channels and the regulation of inflammatory gene expression, extending beyond classical inflammasome and cytokine activation to include transcriptional control of inflammatory responses. Furthermore, through the calcium signaling in lysosomes (mediated by TRPMLs or TPCs), immune cells control exocytosis of lysosomes, phagocytosis, the presentation of antigens, motility, chemotaxis, and the release of cytokines/chemokines among other things. Thus, the lysosome stands at the crossroads of degradation, metabolic sensing, and immune signaling a versatile organelle with multifaceted influence on immune cell behavior (23).

### Dysfunction of lysosome ion channel regulation and inflammation

**Figure 1:**
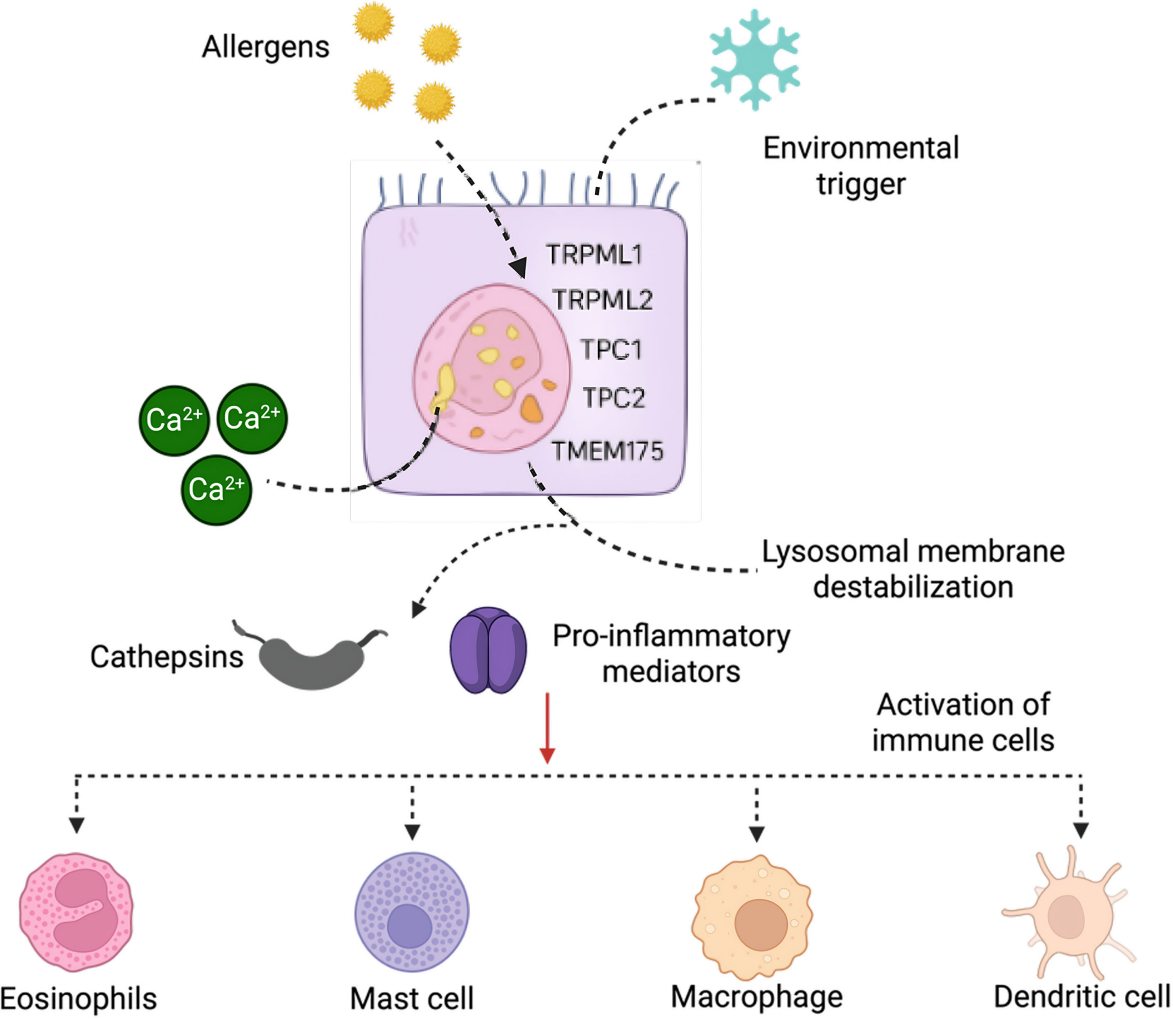
Pathophysiology of airway inflammation triggered by lysosomes. Nasal epithelial cells internalize environmental triggers and airborne allergens, which cause membrane destabilization, ion channel activation (TRPMLs, TPCs, TMEM175), and lysosomal stress. When the NLRP3 inflammasome is activated by cathepsin release, Ca2+ flow, and ROS, caspase-1 is activated and IL-1β and IL-18 are processed. These cytokines contribute to airway allergy disease by initiating downstream inflammatory cascades that include eosinophil recruitment, mast cell activation, mucus hypersecretion, and disruption of the epithelial barrier.

Lysosomal ion channels are so important that their dysregulation due to genetic alterations, environmental impacts, and other disturbances can cause lysosomal dysfunction, autophagy impairment, trafficking errors, ion homeostasis disruptions, and immune signaling changes (24). In the context of inflammation, such dysfunction may alter immune responses in several ways. Inadequate clearance of inflammasomes or damaged organelles can promote chronic inflammation, while unstable lysosomal membranes may release cathepsins and other lysosomal contents, thereby triggering inflammasome activation. Additionally, aberrant ion flux may dysregulate intracellular signaling cascades and further amplify inflammatory responses. Indeed, lysosomal ion-channel dysregulation has been implicated in various pathological conditions neurodegenerative diseases, storage disorders, autoimmune diseases, and inflammation-related diseases (25) (**Figure 1**). In immune cells, impaired lysosomal function may disturb antigen processing and presentation, cytokine secretion, and immune cell metabolism potentially contributing to dysregulated inflammation or reduced pathogen clearance (26).

### Molecular mechanisms of lysosome-triggered inflammation in nasal and upper airways

**Figure 2:**
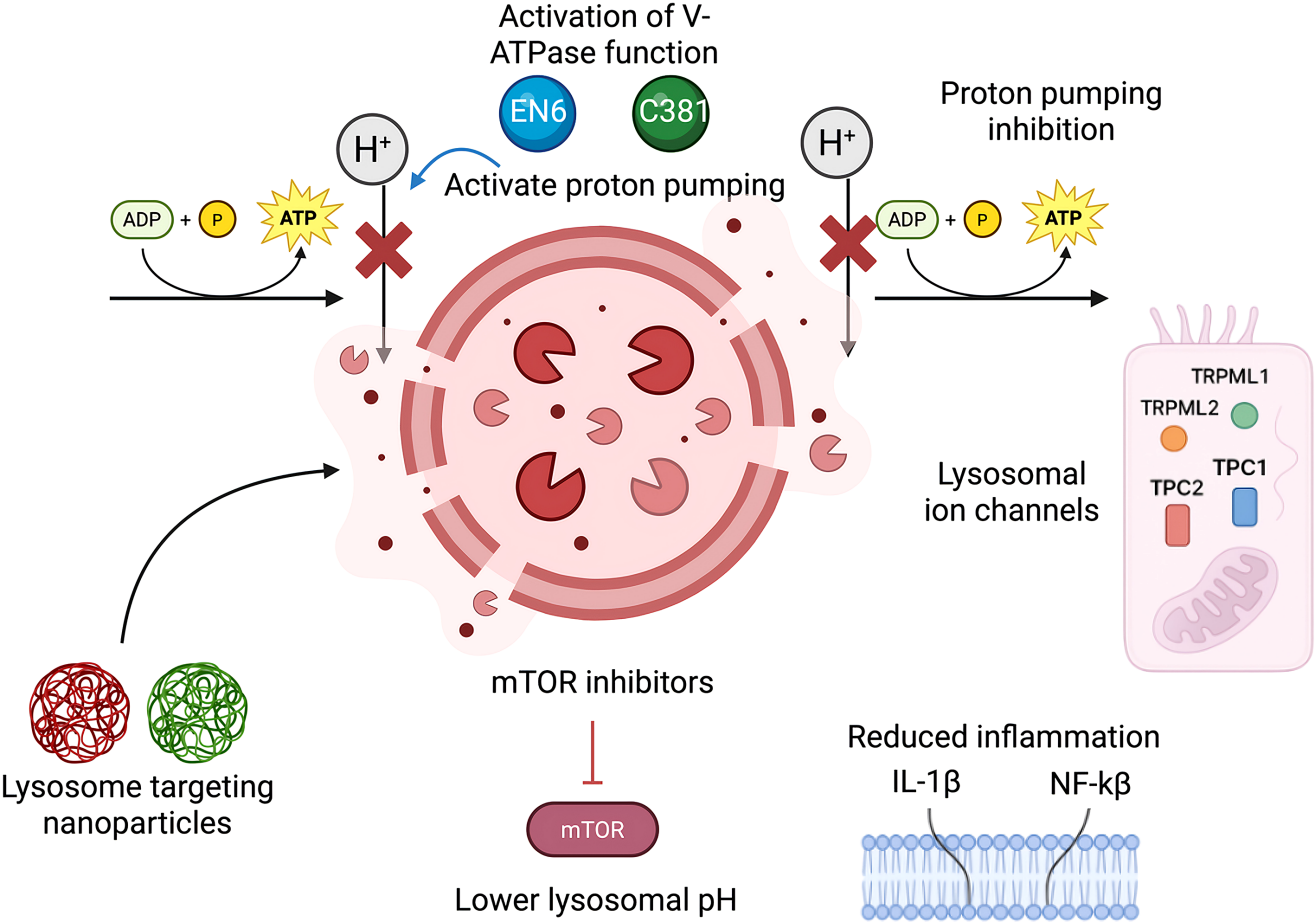
Mechanism of therapeutic targeting of inflammatory pathways triggered by lysosomes. The multi-level therapeutic targeting of lysosome-triggered inflammatory signaling in upper-airway and nasal allergy illness is illustrated in this image. Lysosomal dysfunction, which is characterized by abnormal activation of lysosomal ion channels, excessive Ca2^+^ release, and disruption of lysosomal membrane integrity, is induced in airway epithelial and immunological cells by allergen exposure and environmental stresses. These processes encourage oxidative stress, cytosolic cathepsin leakage, and NLRP3 inflammasome activation, which leads to caspase-1-dependent maturation and production of IL-1β and IL-18. Inflammasome inhibitors and cytokine-neutralizing biologics suppress downstream inflammatory signaling; small-molecule modulators normalize lysosomal ion flux and restore intracellular homeostasis; gene-based strategies, such as siRNA and CRISPR-Cas9, decrease pathological expression of lysosomal channels and inflammasome components; and nanoparticle-based delivery systems improve tissue-specific targeting and therapeutic efficacy. When combined, these tactics lessen the generation of inflammatory cytokines, decrease the recruitment of immune cells, maintain the integrity of the epithelial barrier, and lessen persistent airway inflammation.

**Figure 3:**
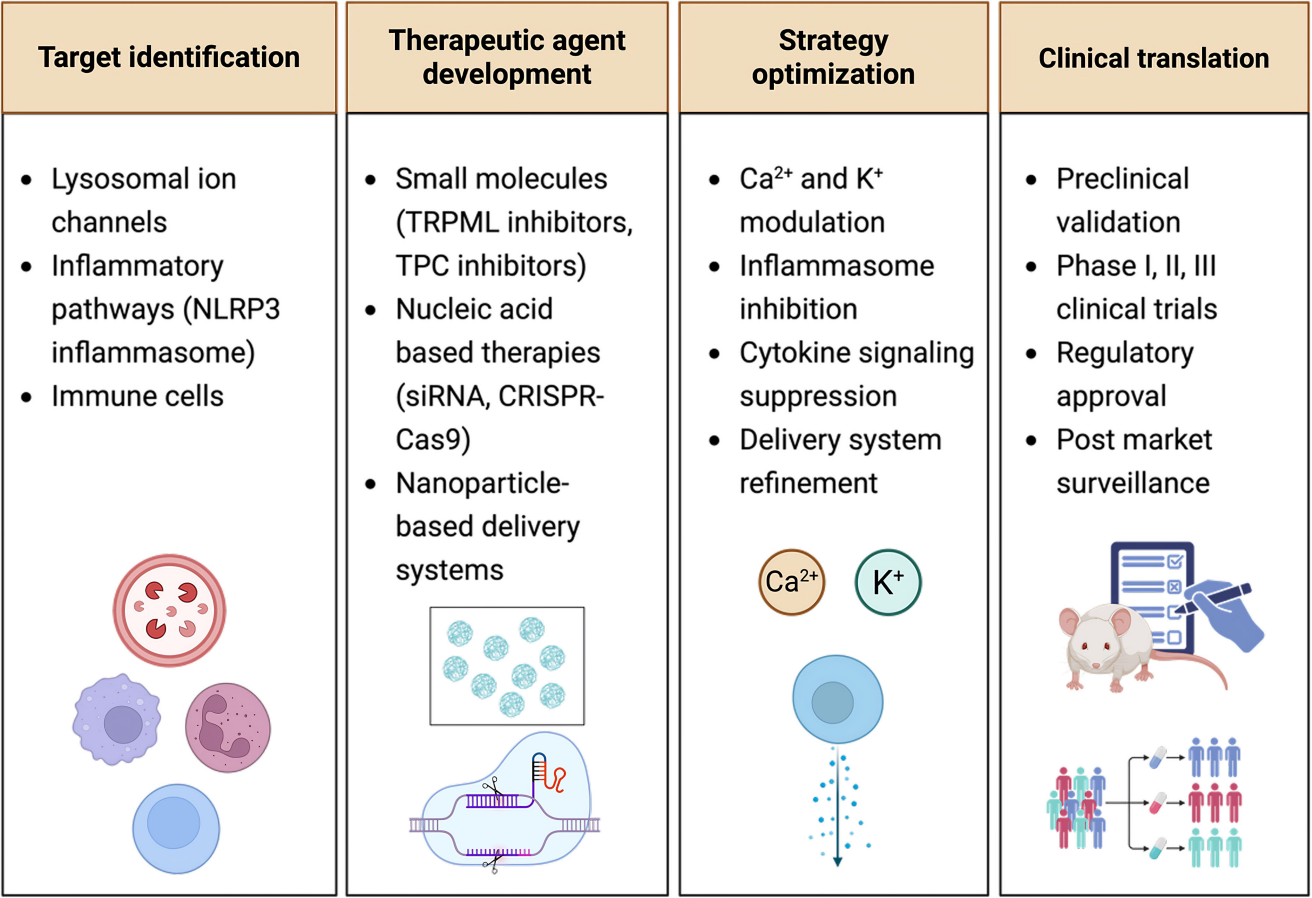
Lysosomal channel-targeted therapy mechanism. By controlling ion flow and lysosomal stability in immunological and airway epithelial cells, lysosomal channel-targeted treatments alter intracellular inflammatory signaling. Excessive activation of lysosomal ion channels, including TRPML1, TRPML2, TPC1, TPC2, and TMEM175, causes aberrant Ca^2+^ and K+ flux, destabilization of the lysosomal membrane, and cathepsin release in the cytosol under pathological conditions. Pro-inflammatory cytokines IL-1β and IL-18 are secreted as a result of caspase-1-mediated maturation and activation of the NLRP3 inflammasome. Small-molecule TPC inhibitors suppress excessive lysosomal Ca^2+^ release and downstream inflammasome activation; TRPML1 agonists improve autophagic flux, restore physiological Ca^2+^ signaling, and facilitate the removal of damaged organelles; gene-based strategies, such as siRNA and CRISPR-Cas9, decrease aberrant expression of lysosomal ion channels; and nanoparticle-based delivery systems enhance selective targeting to airway epithelial cells. When taken as a whole, these treatments decrease the generation of inflammatory cytokines, restrict the recruitment of immune cells, maintain the integrity of the epithelial barrier, and lessen lysosome-mediated airway inflammation.

Allergic inflammation in the nasal and upper-airway mucosa is a complex process involving multiple cellular and molecular pathways. Recent evidence highlights lysosomes as central regulators of these inflammatory responses, particularly through ion-channel-mediated signaling, inflammasome activation, and crosstalk with other immune pathways (27, 28) (**Figures 2**, **3**).

### Role of lysosomes in airway epithelial cells

Airway epithelial cells serve as the first line of defense against allergens and environmental stimuli (29). Lysosomes in these cells are involved in processing internalized allergens, regulating antigen presentation, and controlling the secretion of pro-inflammatory cytokines and chemokines. Lysosomal dysfunction or destabilization may result in the release of cathepsins and other hydrolases into the cytosol, which can activate signaling pathways, including the NLRP3 inflammasome. This process promotes the maturation and secretion of IL-1β and IL-18, contributing to epithelial barrier dysfunction and the recruitment of immune cells to sites of allergen exposure (**Table 1**) (35, 36).

**Table 1:** Environmental and endogenous triggers of lysosome-triggered inflammatory channels in upper-airway allergic disease.

Trigger	Lysosomal mechanism	Affected cells	Key outcomes	Ref.
Environmental				
Pollen	Endocytosis → lysosomal destabilization → cathepsin release	Nasal epithelial cells, DCs	NLRP3 inflammasome activation, IL-1β/IL-18 secretion, eosinophil recruitment	(30)
Dust mites	Phagocytosis → membrane permeabilization	Epithelial cells, macrophages	Enhanced cytokine release, mucus hypersecretion	(31)
Pollutants	Lysosomal membrane permeabilization, ROS generation	Epithelial cells, immune cells	Oxidative stress, chronic inflammation	(32)
Endogenous				
Microbial components (LPS, viral particles)	Lysosome-TLR crosstalk → inflammasome priming	DCs, macrophages	Increased pro-IL-1β, Th2 polarization	(20)
Oxidative stress (ROS)	Membrane damage → ion flux disruption	Epithelial cells, mast cells	NF-κB activation, epithelial barrier dysfunction	(33)
Mechanical/chemical stress	Ca²^+^/K^+^ flux dysregulation	Nasal epithelial cells	Amplified inflammasome activation	(34)

In upper-airway allergic models, environmental allergens such as dust mites and pollen have been shown to induce lysosomal destabilization following endocytosis or phagocytosis by epithelial cells, DCs, and macrophages, leading to inflammasome activation, cytokine release, eosinophilic inflammation, and mucus hypersecretion (30, 31). Similarly, particulate matter and pollutants can induce lysosomal membrane permeabilization and ROS production in epithelial and immune cells, promoting oxidative stress and persistent inflammation (32).

In contrast, mechanisms involving LPS and viral particles are primarily supported by broader inflammatory and immune-response studies rather than direct upper-airway allergic models. These microbial components activate lysosome-mediated TLR signaling in macrophages and DCs, enhancing Th2 polarization, pro-IL-1β production, inflammasome priming, and antigen presentation (20). Likewise, endogenous oxidative, mechanical, or chemical stress may disrupt lysosomal membrane integrity or ion fluxes in mast cells and epithelial cells, contributing to IL-1β/IL-18 production, NF-κB activation, epithelial barrier dysfunction, local edema, and immune cell recruitment (33, 34).

### NLRP3 inflammasome activation

The NLRP3 inflammasome is a cytosolic multiprotein complex that acts as a sensor for cellular stress and danger signals. Activation occurs in response to lysosomal destabilization, often triggered by particulate allergens or reactive oxygen species (ROS) (37). The NLRP3 inflammasome is activated, and subsequently, ASC and caspase-1 are recruited, resulting in the conversion of pro-IL-1β and pro-IL-18 into their active forms (38). This mechanism in nasal epithelial cells intensifies local inflammation and causes edema, mucus hypersecretion, and hyperresponsiveness. Moreover, NLRP3 activation may trigger pyroptosis, an inflammatory cell death process that further increases the inflammation of the affected tissue (39) (**Table 2**). After endocytosis, allergens such dust mites and pollen cause lysosomal destabilization, which results in cathepsin release, NLRP3 inflammasome activation, and IL-1β/IL-18 secretion. This causes nasal mucosal inflammation, oedema, and mucus hypersecretion (40). After phagocytosis, particulate debris and pollutants cause lysosomal membrane permeabilization, which triggers caspase-1 activation and pyroptosis, aggravating sinusitis and chronic rhinitis (41).

**Table 2:** NLRP3 inflammasome activation pathways in upper-airway allergic inflammation.

Trigger	Lysosomal mechanism	Downstream effects	Clinical relevance	Ref.
Allergens (pollen, dust mites)	Endocytosis → destabilization → cathepsin release	NLRP3 activation, IL-1β & IL-18 secretion	Nasal mucosa inflammation, edema, and hypersecretion	(40)
Particulate matter/pollutants	Phagocytosis → membrane permeabilization	Caspase-1 activation, pyroptosis	Exacerbation of chronic rhinitis and sinusitis	(41)
ROS (oxidative stress)	Lysosomal membrane destabilization	IL-1β/IL-18 maturation, NF-κB activation	Enhances epithelial barrier dysfunction	(42)
Microbial (LPS, viruses)	Lysosome-mediated TLR signaling	Inflammasome priming, pro-IL-1β synthesis	Exacerbates secondary allergic inflammation	(43)
Physical stress	Lysosomal ion flux disruption (Ca²^+^, K^+^)	NLRP3 assembly, cytokine secretion	increased immune cell recruitment in nasal mucosa	(44)

Lysosomal membrane instability brought on by oxidative stress-induced ROS promotes IL-1β/IL-18 maturation and NF-κB signalling, which eventually contributes to epithelial barrier failure (42). Secondary allergic inflammation may be exacerbated by microbial components, such as LPS and viral particles, which trigger lysosome-mediated TLR signaling, inflammasome priming, and enhanced pro-IL-1β production (43). When lysosomal Ca2+ and K+ fluxes are disrupted by mechanical or chemical stress, NLRP3 inflammasome assembly, cytokine release, and increased immune cell recruitment occur in the nasal mucosa (44).

### Ion-channel mediated lysosomal signaling

Among the lysosomal channels, TRPML and TPCs have the greatest impact on regulating intracellular calcium and other ion movements, which are essential for the activation of the inflammasome and the subsequent release of cytokines. In airway epithelial and resident immune cells, the activation of downstream pathways, such as NF-κB and MAPK, which control the transcription of pro-inflammatory genes, has been linked to the calcium release from lysosomes through TRPML1 channels. When the activity of the lysosomal ion channels is altered, the inflammatory responses can be amplified, thus leading to the development or maintenance of chronic or severe allergic conditions (45, 46).

### Crosstalk with immune cells

**Figure 4:**
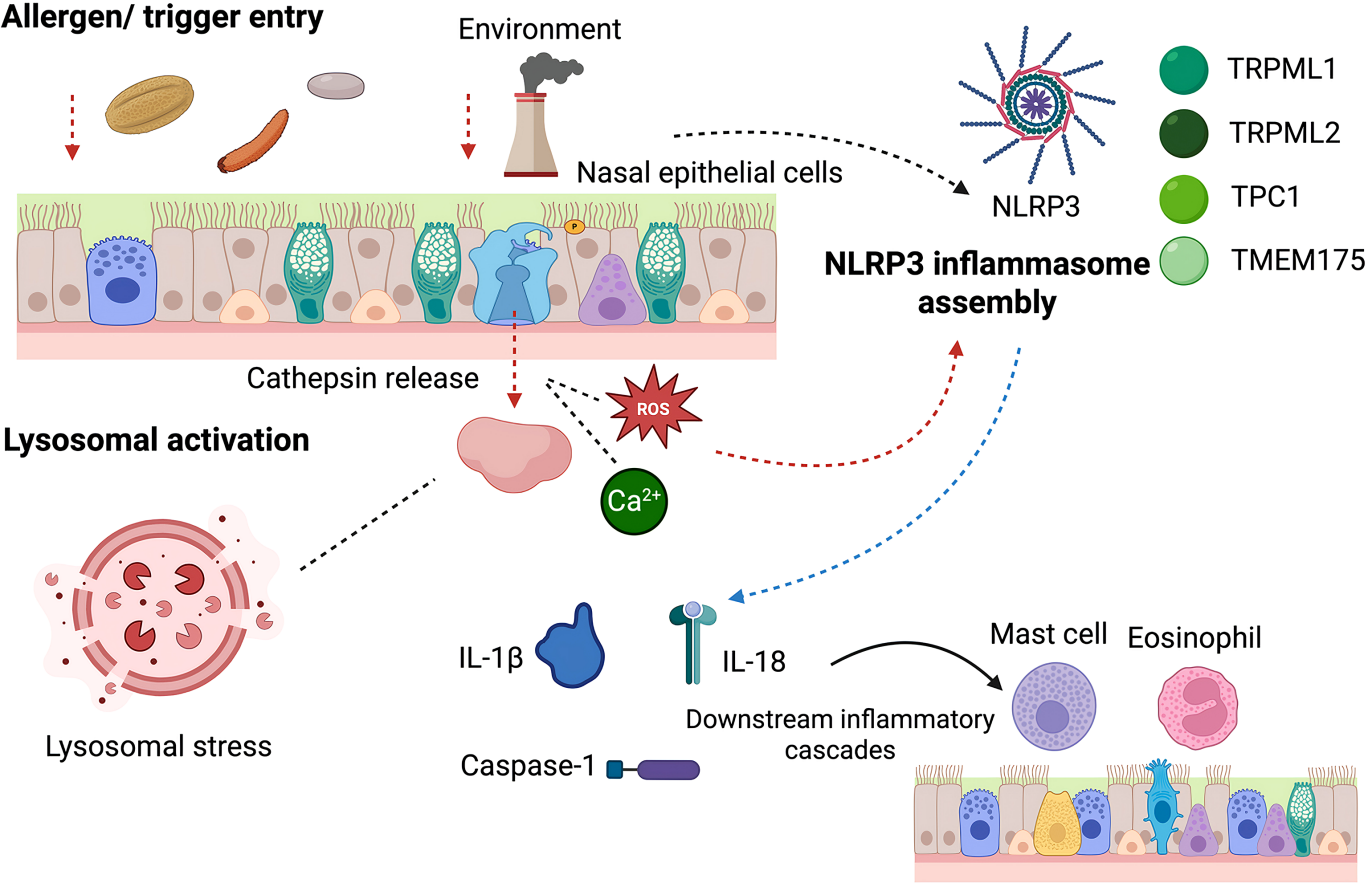
Lysosomal channel interaction with immune cells in allergic inflammation of the upper respiratory tract. The dynamic interplay between immune cells and lysosomal ion channels during upper-airway allergic inflammation is depicted in this picture. Activation of lysosomal channels, such as TRPML1, TRPML2, TPC1, TPC2, and TMEM175, controls intracellular Ca^2+^ flux and lysosomal stability in airway epithelial cells. Cathepsins and pro-inflammatory mediators are released when lysosomal membrane permeabilization is facilitated by dysregulated channel activity. Immune cells including eosinophils, mast cells, macrophages, and DCs are recruited and activated more easily thanks to these signals. By promoting antigen processing, inflammasome activation, and cytokine production in antigen-presenting cells, lysosomal Ca^2+^ signaling connects innate immune detection to adaptive immunological responses. Mast cell degranulation and eosinophil activation intensify inflammatory cascades, leading to mucus hypersecretion, breakdown of the epithelial barrier, and persistent allergic inflammation in the upper respiratory tract.

Lysosomal signaling in epithelial cells is not a singular event. Cytokines and chemokines that are released in a lysosome-dependent manner not only recruit but also activate mast cells, eosinophils, and DCs (47). For mast cells, lysosomal degranulation can cause an increase in histamine and protease release that will imperceptibly raise vascular permeability and cause edema. Eosinophils respond to lysosome-mediated signals by producing ROS and cytokines, which further perpetuate inflammation. DCs internalize allergens via lysosome-rich endosomes and present them to T cells, linking innate and adaptive immunity (48). This multicellular interplay creates a self-reinforcing inflammatory loop in allergic upper-airway disease (**Figure 4**).

### Environmental triggers and lysosomal activation

Lysosomes of cells in the environment such as pollen, dust mites, and particulate matter can ruin their stability directly or by the action of ROS being generated. Furthermore, pollutants and the components of microorganisms can cause the activation of TLRs and hence the extinction of lysosome-dependent inflammasome assembly, indicating that lysosomes are playing the role of central integrators of both external and internal danger signals. Although this integration is essential for maintaining a rapid yet regulated inflammatory response, excessive or chronic lysosomal activation can be detrimental, leading to persistent inflammation, tissue damage, and the progression of allergic disease (9, 49, 50).

### Lysosomal ion channels: disease relevance

Lysosomal ion channels have emerged as important regulators of intracellular signaling pathways involved in immune responses and inflammatory diseases, including nasal and upper-airway allergic inflammation (51, 52). In airway epithelial and immune cells, these channels regulate Ca²^+^ and other ion fluxes that influence inflammasome activation, cytokine production, and immune cell recruitment.

TRPML1-mediated lysosomal Ca²^+^ release has been implicated in NLRP3 inflammasome activation, leading to increased secretion of IL-1β and IL-18 in epithelial and immune cells (5). TRPML2 and TRPML3 further regulate endolysosomal trafficking processes, including cytokine transport and lysosome–endosome fusion, thereby influencing antigen processing and immune cell activation (45, 53). Two-pore channels (TPC1 and TPC2) modulate lysosomal Na^+^ and Ca²^+^ signaling; TPC2 has been associated with enhanced cytokine production and inflammatory amplification, while TPC1 contributes to NF-κB activation in epithelial cells (54, 55).

Other channels, including TMEM175, regulate lysosomal stability and autophagy, indirectly modulating inflammatory signaling pathways (56). Chloride channels further support inflammasome activation by maintaining ionic balance during Ca²^+^ release (57). Collectively, dysregulation of lysosomal ion channels can amplify epithelial barrier dysfunction, cytokine release, and immune cell recruitment in allergic airway disease. Understanding these mechanisms provides a foundation for identifying potential therapeutic targets in upper-airway inflammatory disorders (**Table 3**).

**Table 3:** Key lysosomal ion channels and their functions in upper-airway allergic inflammation.

Ion channel	Primary function	Role	Ref.
Endo-lysosomal			
TRPML2	Cytokine trafficking, lysosome-endosome fusion	Modulates dendritic cell cytokine release, contributes to immune cell recruitment	(53)
TRPML3	Phagosome-lysosome fusion	Influences mast cell degranulation and antigen processing	(45)
TPC1	Na^+^/Ca²^+^ flux, membrane potential regulation	Modulates Ca²^+^ signaling in epithelial cells, triggers NF-κB activation	(54)
TPC2	Na^+^/Ca²^+^ flux, lysosome trafficking	Facilitates cytokine secretion, enhances local inflammation	(55)
Lysosomal			
TRPML1	Ca²^+^ release, lysosomal exocytosis	Activates NLRP3, mediates IL-1β and IL-18 secretion	(5)
TMEM175	K^+^ channel, maintains membrane potential	Supports lysosomal function; indirectly regulates inflammation	(56)
Cl^-^ channels	Charge balance for ion flux	Regulates Ca²^+^ release and inflammasome activation	(57)

### Transient receptor potential mucolipin channels

The TRPML family consists of three main members: TRPML1, TRPML2, and TRPML3. These non-selective cation channels are permeable to Ca²^+^, Fe²^+^, and Zn²^+^ and are localized predominantly on lysosomal and endolysosomal membranes (45). The most studied member, TRPML1, regulates lysosomal exocytosis, autophagy, and trafficking. In airway epithelial cells, TRPML1-mediated Ca²^+^ release contributes to NLRP3 inflammasome activation and secretion of IL-1β and IL-18 (58). Dysfunctional TRPML1 activity has been linked to exaggerated inflammatory responses in allergic airway models. TRPML2 and TRPML3 are implicated in endolysosomal trafficking and immune cell function. TRPML2 is expressed in DCs and macrophages and facilitates cytokine trafficking, whereas TRPML3 participates in phagosome-lysosome fusion (59).

### Two-pore channels

TPCs including TPC1 and TPC2, are cation-selective channels found on endolysosomal membranes. They primarily mediate Na^+^ and, in some contexts, Ca²^+^ release. TPCs contribute to lysosomal pH regulation, membrane potential maintenance, and lysosomal trafficking (60). In allergic airway inflammation, TPC-mediated Ca²^+^ signaling can activate downstream kinases and transcription factors such as NF-κB and MAPK, enhancing pro-inflammatory cytokine production. Moreover, besides TPCs activating lysosome-to-plasma membrane fusion, they also allow the secretion of lysosomal contents thereby increasing the local inflammation (16).

### Potassium and chloride channels

Potassium channels, like TMEM175 and BK-like channels, take an active part in the maintenance of the lysosomal membrane potential and the regulation of ion homeostasis. In addition to this, chloride channels help keep the charge in balance during ion flux and thus maintain the proper release of Ca²^+^ and Na^+^ (61, 62). Indirectly, these channels are affecting the activation of inflammasomes and the autophagic flux along with the lysosomal trafficking in both the epithelial and immune cells.

### Functional implications in upper-airway allergic disease

Lysosomal ion channels are actively involved throughout the progression of allergic inflammation. With the help of the lysosomal ion channels coordinating the intracellular signaling regulation, the inflammatory and immune processes impacted by those channels were plethora. TRPML1 or the two-pore channel-mediated calcium release can start the building of the NLRP3 inflammasome, which in turn does the cytokine maturation, and consequently, the amplification of the inflammatory responses takes place (58). Also, ion translocation through the lysosomal membranes is involved in the process of lysosome-plasma membrane fusion, thus enabling pro-inflammatory mediator secretion. Moreover, proper lysosomal ion channel function is essential for maintaining lysosomal pH and intracellular trafficking, which supports efficient autophagy and the clearance of damaged organelles, thereby helping to limit chronic or excessive inflammation (63). In immune cells such as macrophages, DCs, and mast cells, lysosomal ion channels are involved in the regulation of key functions, including chemotaxis, phagocytosis, and mediator release, highlighting their importance in immune regulation. If any of these channels fall out of regulation, the result will be a persistent inflammation scenario coupled with epithelial barrier dysfunction, and hence, more intense allergic responses; thus, they are viewed as potential points for pharmacological intervention (64).

### Therapeutic approaches targeting lysosome-triggered channels

**Figure 5:**
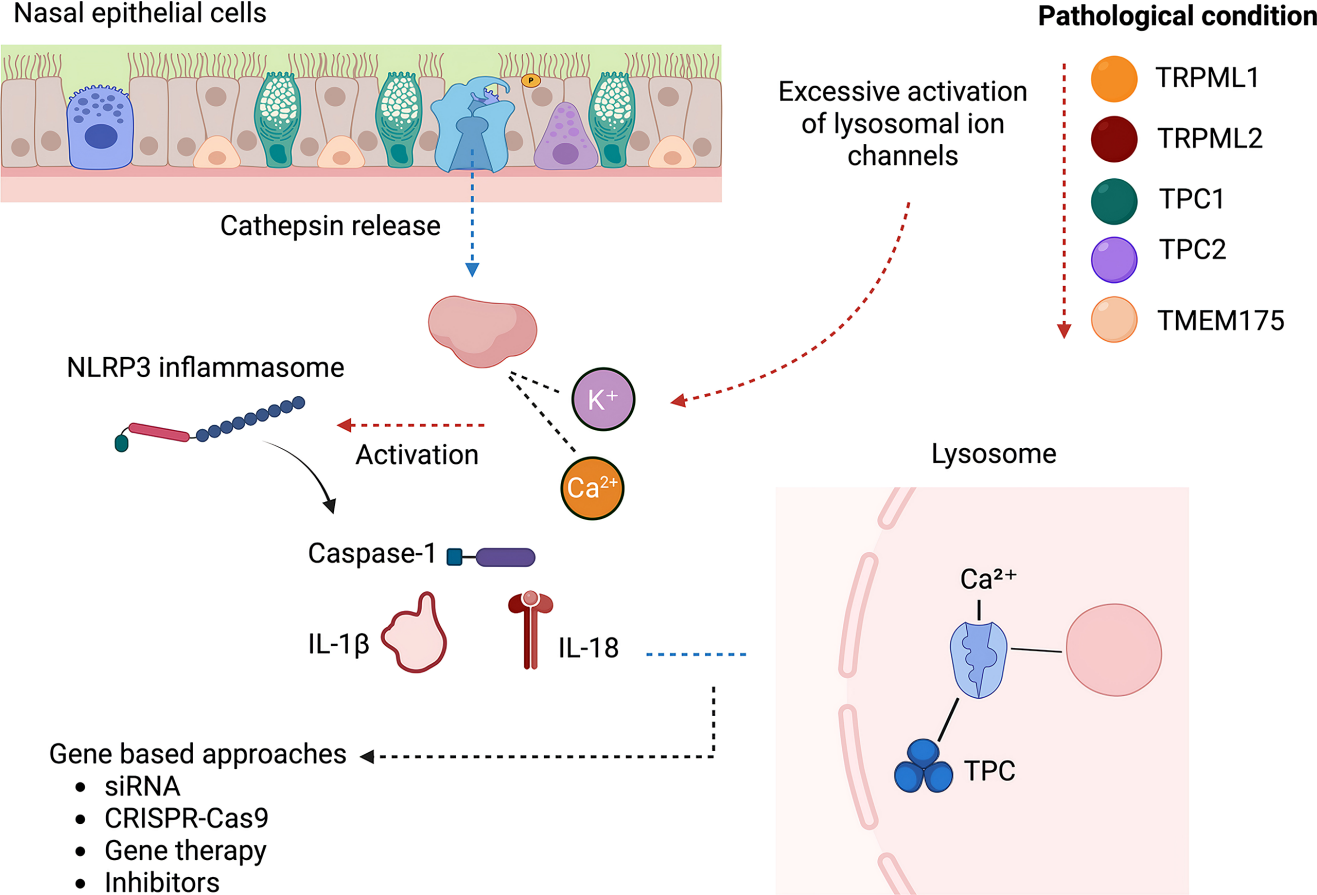
A suggested treatment plan for upper-airway allergy illness that focuses on lysosome-triggered inflammatory pathways. An image depicts a sequential therapeutic path from the discovery of lysosome-targeted medications to their clinical usefulness in treating nasal and upper-airway allergies. The first step of the schematic is to discover the key molecular targets that are, among others, the lysosomal ion channels (TRPML1, TRPML2, TPC1, TPC2, and TMEM175) and the NLRP3 inflammasome-related pathways, which are known to be prevalent in both airway epithelial and immunological cells. Following that, it comes the second phase of the therapeutic agents’ development comes as it now includes the small-molecule channel modulators, nucleic acid-based treatments (siRNA and CRISPR-Cas9), and nanoparticle-based delivery systems that are designed to enhance tissue selectivity and improve therapeutic efficacy. The third step is targeting treatment methods, e.g., via improving the drug delivery systems, inflammasome activation suppression, cytokine signaling downregulation, and manipulating lysosomal calcium and potassium ion flux. The last stage of clinical translation includes preclinical validation, staged clinical trials, regulatory approval, and post-marketing surveillance. All in all, this roadmap presents an integrated precision medicine strategy meant to gain long-term control over upper-airway allergy-related diseases, diminish airway inflammation, and restore the epithelial barrier’s integrity.

Targeting lysosome-triggered inflammatory pathways offers a novel therapeutic strategy for managing nasal and upper airway allergic conditions, particularly allergic rhinitis and related inflammatory disorders. By modulating lysosomal ion flux, inflammasome activation, and downstream cytokine production, these interventions aim to reduce local inflammation, limit epithelial damage, and restore immune homeostasis (6) (**Figure 5**; **Table 4**). Inflammasome-driven inflammation in the nasal mucosa is linked to endogenous and external stimuli by lysosomal dysfunction. In epithelial and immune cells, allergens, pollutants, microbial components, oxidative, mechanical, and chemical stress can cause lysosomal destabilization, membrane permeabilization, or ion flux dysregulation. This can result in NLRP3 inflammasome activation, caspase-1 signaling, IL-1β/IL-18 release, NF-κB activation, epithelial barrier dysfunction, mucus hypersecretion, and chronic inflammation (20, 30–34, 40–44). Lysosomal ion channels, such as TRPML1-3, TPC1/2, TMEM175, and Cl^-^ channels, which regulate Ca2^+^, Na^+^, K^+^, and Cl^-^ fluxes, lysosomal trafficking, cytokine release, autophagy, and immune cell recruitment, tightly regulate these activities (5, 45, 53–57).

**Table 4:** Therapeutic agents targeting lysosome-triggered channels in upper-airway allergic inflammation.

Therapeutic approach	Target	Mechanism of action	Preclinical/clinical evidence	Ref.
Small-molecule agonists (ML-SA1)	TRPML1	Activates lysosomal Ca²^+^ release, enhances autophagy	Reduced NLRP3 activity and cytokine secretion in murine AR models	(54)
Small-molecule inhibitors (Tetrandrine, Ned-19)	TPC1/TPC2	Inhibits excessive Ca²^+^ signaling	Decreased IL-1β production and airway inflammation	(65)
Monoclonal antibodies (anti-IL-1β, anti-IL-18)	Downstream cytokines	Neutralizes pro-inflammatory cytokines	Reduced nasal edema and eosinophilic infiltration (animal models	(66)
RNA interference (siRNA)	TRPML1, TPCs	Gene silencing, reduce lysosomal Ca²^+^ flux	Attenuated NLRP3 activation and mediator release (*in vitro*/*in vivo*)	(67)
CRISPR-Cas9 (Gene editing)	TRPML1, TPCs	Knockout for modulation of channel expression	Improved epithelial barrier integrity; decreased inflammasome activation	(27, 68)

In preclinical *in vitro* and animal models, targeting these pathways using TRPML1 agonists, TPC inhibitors, cytokine-neutralizing antibodies, or gene-based approaches such as siRNA and CRISPR-Cas9 has been shown to reduce inflammasome activation, cytokine release, and airway inflammation. These findings support the mechanistic relevance of lysosomal ion channels and downstream inflammasome signaling in allergic airway inflammation; however, they do not yet establish clinical efficacy or applicability in human upper-airway allergic disease (27, 54, 65–68).

### Small-molecule modulators

Small-molecule inhibitors and activators of lysosomal ion channels have shown promise in preclinical studies. TRPML1 agonists, such as ML-SA1, can restore lysosomal calcium release and promote autophagic clearance, reducing inflammasome-mediated cytokine secretion (69). On the other hand, calcium signaling that causes inflammation in airway epithelial cells can be prevented by the use of TPC inhibitors like tetrandrine or Ned-19. These substances are capable of selectively modulating lysosome-related pathways without the risk of broadly suppressing the immune system (65).

### Biologics and targeted proteins

New monoclonal antibodies together with protein-based therapies, which indirectly target lysosomal signaling, are getting recognized as complementary agents to standard treatments. One example is the use of neutralizing antibodies against IL-1β or IL-18, which can reduce the downstream inflammatory effects resulting from lysosome-mediated inflammasome activation. Moreover, lysosomal stabilization or cathepsin inhibition are other strategies that could potentially lead to the reduction of epithelial injury and the release of proinflammatory mediators (70, 71).

### Gene-based approaches

Advances in gene-editing technologies, including CRISPR-Cas9 and RNA interference, have created new opportunities for the targeted modulation of lysosomal ion channels and inflammasome components. TRPML1 or TPC expression knockdown in airway epithelial or immune cells results in the secretion of less IL-1β and the reduction of inflammatory responses in experimental allergic airway models. The precision offered in controlling the signaling pathways of lysosomes might also lead to the development of personalized treatments (72, 73).

### Preclinical and clinical evidence

Results from multiple preclinical studies suggest that targeting lysosomal ion channels may have therapeutic potential in allergic airway inflammation. For example, in murine models of allergic rhinitis, pharmacological inhibition of TPC2 reduced nasal mucosal inflammation, decreased eosinophil infiltration, and suppressed Th2 cytokine production (74). Similarly, activation of TRPML1 has been associated with enhanced autophagic activity and reduced NLRP3 inflammasome activation in experimental models. Although these findings provide important mechanistic insights, they are derived exclusively from preclinical studies, and human clinical trials have not yet been established. Therefore, their translational relevance to human upper-airway allergic disease remains to be confirmed (75).

### Integration with conventional therapies

Lysosome-directed therapies may be considered as adjunctive strategies alongside established treatments such as antihistamines, corticosteroids, or allergen immunotherapy. Through selective modulation of intracellular inflammatory pathways, these approaches may potentially contribute to reducing the required doses of systemic medications and, consequently, may help limit adverse effects while potentially improving overall therapeutic control. However, these outcomes remain hypothetical and require further experimental and clinical validation. Moreover, combination strategies may simultaneously target upstream signaling pathways triggered by lysosomal activation and downstream effector responses, such as cytokine production and histamine release (76).

## Future perspectives

Future approaches may involve nanoparticle-based delivery systems designed to selectively target lysosomal channels in airway epithelial and immune cells, with the aim of improving drug localization while potentially reducing systemic exposure. In addition, patient-stratified strategies based on genomic or proteomic profiling may help identify subgroups more likely to benefit from lysosome-targeted interventions. Overall, targeting lysosomal ion channels represents a potentially promising and emerging direction in the management of allergic upper-airway diseases; however, its clinical applicability remains to be established in future translational and clinical studies.

## Challenges and limitations in therapeutic targeting

Even though targeting lysosome-triggered inflammatory pathways is a very good strategy to treat nasal and upper-airway allergies, there are still many challenges that need to be solved before such therapies can be applied on a large scale. It is essential to realize these limitations to develop interventions that are safe, effective and translatable to the clinic.

### Off-target effects

Lysosomal ion channels are not only found in airway and immune cells but also present in various other cells like neurons, liver, and heart cells. The global alteration of these channels might accidentally influence the operation of lysosomes in the tissues that are not the intended target, which may result in unwanted outcomes like neurotoxicity, modified metabolism, or cardiac impairment. Therefore, the use of small-molecule modulators that are very selective, along with very careful dosing strategies, is crucial in the reduction of off-target toxicity (77, 78).

### Drug delivery challenges

The nasal and upper airway environment presents unique challenges for effective drug delivery. Mucociliary clearance, enzymatic breakdown, and poor epithelial penetration are just some of the mechanisms that can reduce the effectiveness of lysosome-targeted therapies (79). Although systems that use either nanoparticles or aerosols might lead to better local delivery, they still require optimization to make sure that stability, controlled release, and low immunogenicity are all attained (80, 81).

### Resistance and adaptation

Continuous use of pharmacological inhibitors may lead to the development of cellular compensatory mechanisms that reduce therapeutic efficacy (82). For instance, continuous inhibition of TRPML1 or TPC channels can result in the upregulation of other ion transporters or the activation of inflammatory pathways (45). This type of adaptive response can limit the efficacy of the treatment in the long run and may lead to the need for combination therapies or intermittent dosing as a solution.

### Complexity of lysosomal networks

Lysosomes are the key players in complex intracellular signaling networks that are responsible for the regulation of processes such as autophagy, apoptosis, inflammasome activation, and metabolic sensing. The alteration of one factor can lead to the disruption of several pathways, which might sometimes have unpredictable consequences. The creation of a very thorough understanding of the lysosomal networks on a mechanistic level will be necessary if side effects have to be anticipated and managed (83, 84).

### Translational and clinical barriers

Preclinical research has provided strong proof-of-concept evidence; however, translating these findings from animal models to humans remains highly challenging. Differences in lysosomal channel expression, immune responses, and airway physiology between animal models and humans may significantly influence treatment efficacy and clinical outcomes. Furthermore, the variation in allergic airway diseases, consisting of different allergen triggers, different levels of severity, and different patient genetics, makes it even harder to classify patients and design trials accordingly (84).

### Regulatory and safety considerations

On the other hand, the gene-based methods and the cutting-edge biologics that aim at lysosomal pathways are facing an even stricter scrutiny from the regulatory bodies due to the long-term safety, off-target genome editing, and immune reactions that are concerns (85). The execution of comprehensive preclinical toxicity studies followed by clinical trials with careful monitoring is the only way to assure safety and efficacy. However, these hurdles are being swept away by the ongoing research in delivery, selective modulators, and personalized treatment strategies which are opening the door to the therapeutic capability of lysosome-targeted interventions (86).

### Emerging strategies and future directions

Recent advances in cell biology, nanotechnology, and molecular therapeutics have opened new avenues for targeting lysosome-mediated inflammatory pathways in nasal and upper airway allergic conditions, particularly allergic rhinitis and related inflammatory disorders. The new strategies that are being developed are intended to raise the ability, sharpen the accuracy, and reduce the systemic side effects.

### Nanoparticle-based delivery systems

The use of nanoparticles as carriers for small-molecule modulators, biologics, or nucleic acids makes it possible to deliver the drugs directly to the airway epithelial and immune cells. Such systems protect the therapeutic agents against enzymatic degradation, provide a gradual release, and help to get through the mucus barrier (87). Lipid-based nanoparticles and polymeric nanocarriers have been able to deliver ion-channel modulators very efficiently and carry out the same with siRNA constructs to respiratory epithelial cells in preclinical models, thus significantly reducing local inflammation and at the same time limiting the exposure to the whole body (88).

### Gene editing and RNA interference

The precise targeting of lysosomal ion channels or inflammasome components can be achieved through the use of gene-editing technologies like CRISPR-Cas9 and RNA interference (RNAi). If there is a targeted knockdown of TRPML1 or TPCs in the airway epithelial and immune cells, it may lead to a reduction in lysosome-triggered calcium signaling, NLRP3 inflammasome activation, and the production of cytokines. Such methods are very promising for tailoring therapy, particularly for patients suffering from refractory allergic inflammation or possessing genetic predispositions that affect lysosomal functions (89).

### Combination therapies

Targeting lysosomal pathways in treatment may potentially be used in combination with conventional therapies such as antihistamines, corticosteroids, or allergen immunotherapy. By modulating upstream lysosomal signaling alongside downstream effector responses, such combination strategies may help reduce the required doses of systemic medications and thereby potentially limit associated side effects (90). Furthermore, simultaneous targeting of multiple lysosomal channels or the addition of antioxidant approaches may exert synergistic effects in suppressing airway inflammatory responses.

### Biomarker-guided precision medicine

The identification of biomarkers linked to the disruption of lysosomal channels and triggering of the inflammasome may make it possible to segregate the patients who are more likely to benefit from the targeted therapies. For instance, the upsurge of IL-1β or TRPML1 in nasal epithelial cells might work as predictive indicators for the sensitivity to lysosome-targeted modulators (91). The routine use of transcriptomic and proteomic profiling in medical practice can lead to precision interventions and thus improve treatment outcomes.

### Future research directions

Several problems still arise in spite of the affirming preclinical data:

- Thorough mechanistic research to delineate the entire range of lysosomal ion channel interactions in the airway epithelium and immune cells.
- Development of highly selective and bioavailable modulators suitable for clinical use.
- Optimization of local delivery systems to overcome mucosal barriers and achieve sustained drug concentrations.
- Clinical trials on a large scale are conducted to assess the safety, effectiveness, and long-term consequences in various patient groups.

To summarize, the new strategies reveal the translational capability of lysosome-targeted therapies in the treatment of allergic upper-airway diseases, providing chances to create personalized, fast-acting, and less invasive treatments.

## Conclusion

Lysosome-activated inflammatory pathways play a major role in the development and progression of nasal and upper airway allergic conditions, particularly allergic rhinitis and related inflammatory disorders. Initially, lysosomes were thought to be just degradative organelles, but at present, they are acknowledged as vibrant signaling hubs that control immune responses via ion-channel-mediated calcium release, inflammasome activation, and cytokine secretion. The disturbance in the functioning of lysosomal ion channels, e.g., TRPMLs and TPCs, leads to the breakdown of the epithelial barrier, continuous inflammation, and influx of immune cells, all of which aggravate allergic airway disease.

Therapeutic strategies that focus on these channels are highly promising. Small-molecule modulators, biologics, and gene-based methods all give the possibility of selective modulation of lysosome-dependent pathways, thus decreasing the need for the use of broad immunosuppressive therapies. New technologies, such as nanoparticle-mediated delivery, gene editing, and biomarker-guided precision medicine, will help to increase specificity, efficacy, and safety. The combination of therapies that will involve the use of lysosome-targeted interventions along with conventional treatments may also lead to better clinical outcomes. Nevertheless, there are still challenges to be overcome. Off-target effects, drug delivery barriers, adaptive cellular responses, and individual differences in airway diseases are the reasons why clinical translation is so difficult. To be able to do this, it is essential to conduct mechanistic studies, work on delivery systems optimization, and set up clinical trials with proper design.

In conclusion, targeting lysosome-triggered inflammatory pathways represents a novel and promising approach with significant potential for the management of nasal and upper airway allergic conditions, particularly allergic rhinitis and related inflammatory disorders. Further studies to determine the molecular mechanisms, therapeutic modalities, and patient-specific factors will be vital for turning insights into effective, personalized treatments. By lysosomal biology being integrated into the bigger picture of allergic airway pathophysiology, future therapies would probably yield improved efficacy without increasing the systemic side effects, thus raising patients’ quality of life.
